# Human CTR1 Through the Ages: Milestones and Emerging Roles in Disease and Therapy

**DOI:** 10.3390/biom15121739

**Published:** 2025-12-15

**Authors:** Shahaf Peleg, Lukas Hofmann, Sharon Ruthstein

**Affiliations:** Department of Chemistry and the Institute of Nanotechnology and Advanced Materials, Faculty of Exact Sciences, Bar Ilan University, Ramat-Gan 5290002, Israel

**Keywords:** human CTR1, copper homeostasis, neurodegeneration, cancer, metalloprotein, membrane transporter, cisplatin uptake

## Abstract

Copper transporter 1 (CTR1) is the primary high affinity importer for Cu(I) in eukaryotic cells. CTR1 plays an essential role in maintaining copper homeostasis which is crucial for diverse biological processes. Since its discovery in 1997, research on human CTR1 (hCTR1) has progressed from foundational biochemical characterization to detailed structural and functional elucidation, expanding our understanding of its involvement in human diseases. Here we summarize the current understanding of hCTR1, including its structural features, copper-binding motifs, regulation, trafficking pathways, and roles in disease. We also highlight emerging evidence implicating hCTR1 in cancer, neurodegenerative disorders, and inherited copper metabolism syndromes, emphasizing its potential as a therapeutic target and drug delivery facilitator. Finally, we discuss recent studies and outline future directions, aimed at fully harnessing the biomedical potential of hCTR1.

## 1. Introduction

Copper (Cu) plays a crucial role in fundamental cellular processes. It is involved in cellular respiration, iron oxidation, pigment synthesis, neurotransmitter biosynthesis, antioxidant defense, and connective tissue formation [1,2,3]. Copper is an essential micronutrient required as a prosthetic group for the activity of numerous enzymes that sustain fundamental biological processes. As a cofactor in cuproenzymes such as cytochrome c oxidase, superoxide dismutase, tyrosinase, dopamine β-hydroxylase, and lysyl oxidase, copper supports mitochondrial energy production, antioxidant defense, connective tissue maturation, pigment formation, and neurotransmitter biosynthesis [4]. Conversely, excess of copper is toxic, due to its redox activity, which catalyzes the formation of reactive oxygen species [5,6,7,8]. These dual properties necessitate a tightly regulated system of copper acquisition, intracellular distribution, and export to maintain homeostasis and prevent copper deficiency or excess. Disruptions in copper homeostasis have been associated with severe neurological disorders and various cancer types [6,9,10,11,12,13]. To counteract these effects, cells have evolved intricate regulatory pathways that tightly control intracellular Cu levels.

**Figure 1:**
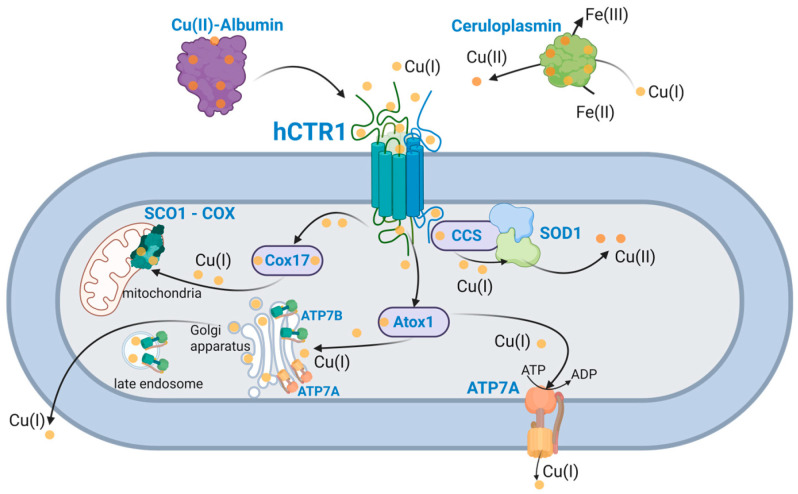
Schematic representation of copper uptake via hCTR1 and intracellular trafficking through copper chaperones.

In humans, Cu follows a well-defined physiological pathway. Dietary copper, primarily in its oxidized Cu(II) form, first enters the bloodstream [14]. Once absorbed, Cu(II) is taken up by the human high-affinity copper transporter 1 (hCTR1), which mediates its cellular import (Figure 1) [15]. hCTR1 was initially discovered in the 1990s due to its sequence homology with the *Saccharomyces cerevisiae* copper transporter [16,17]. Subsequent research established hCTR1 as the central regulator of cellular copper homeostasis. Moreover, later studies uncovered an additional role of hCTR1 in oncology, showing that it also facilitates uptake of platinum-based chemotherapeutic agents, such as cisplatin (cDDP) [18,19].

**Figure 2:**
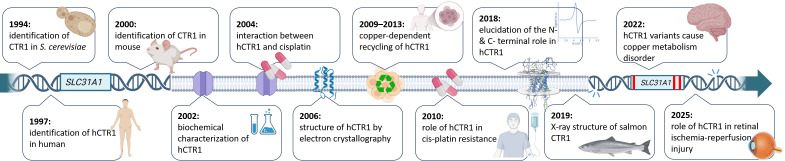
Historical timeline of key discoveries in hCTR1 research, from yeast genetics to human disease relevance.

In less than three decades, research on hCTR1 has evolved from the identification of a copper transporter to the elucidation of a multifunctional protein that acts as both a gatekeeper of copper metabolism and a clinically relevant entry route for modern anticancer agents. This review traces the trajectory of hCTR1 research from its origins in yeast genetics to its current recognition as a critical determinant of human disease and therapeutic response (Figure 2). We then focus on major advances in the molecular and structural characterization of hCTR1 that have elucidated its transport mechanism. And further explore emerging evidence implicating hCTR1 in cancer, neurodegeneration, inherited copper disorders, and its growing significance as a therapeutic target or route for drug delivery. Finally, we highlight unresolved questions and future directions aimed at fully harnessing the biomedical potential of hCTR1.

## 2. Milestones in hCTR1 Research from Discovery to Drug Target

The story of hCTR1 begins not with copper, but with iron. In the late 1980s, researchers studying *Saccharomyces cerevisiae* sought to understand how this unicellular eukaryote acquired iron, an essential but poorly soluble nutrient [20]. At neutral pH, ferric iron Fe(III) has an aqueous solubility of only ~10^−17^ M, far below cellular requirements [21]. Because ferrous iron Fe(II) is considerably more soluble but unstable under aerobic conditions, organisms evolved a mechanism to reduce Fe(III) to Fe(II) at the cell surface prior to uptake [22,23]. In 1990, Dancis and colleagues discovered a yeast mutant lacking ferric reductase activity and showed that this defect also abolished ferric iron uptake. The defective single mutation, termed *fre1-1*, was rescued by the cloned *FRE1* gene [24]. This work provided the first direct genetic evidence that ferric reductase is required for iron assimilation. Importantly, it established a model in which iron uptake required at least two steps: extracellular reduction of ferric to ferrous iron, followed by high-affinity import of Fe(II) across the plasma membrane. A few years later, the same group discovered a new gene, *ctr1*, encoding a high-affinity copper transporter essential for copper uptake. Intriguingly, *ctr1* mutants also displayed a profound defect in iron assimilation, revealing an unexpected mechanistic link between the metabolism of these two essential metals (Figure 1) [17]. This reductive uptake pathway was a change in our understanding of metal assimilation in eukaryotes. It further provided a framework to investigate copper biology, because of the interplay between the high-affinity iron transport system and copper homeostasis. This interplay, unexpected at the time, laid out the groundwork for the discovery of hCTR1. Building on this genetic groundwork, homologues of CTR1 were later identified in mammals, including human CTR1 and mouse CTR1, gene symbol *SLC31A1* (Figure 2) [16]. Subsequently, it was possible to create CTR1 knockout mice models. From which it was possible to deduce the indispensable role of CTR1 in embryonic and neurological development, providing the first direct evidence of its essential physiological role [25,26].

In parallel during the late 90s, Zhou and Gitschier identified the human homolog *SLC31A1*. This gene encodes the 190 amino acid-long protein hCTR1, characterized by its high affinity for copper [16] (Figure 2). Between the years 2000 and 2010 considerable progress was made in the understanding of the protein hCTR1. First, by biochemical characterization and second, by revealing a low-resolution structure of hCTR1 [27,28,29,30,31]. S. Aller and colleagues led by the group of V. Unger reported a 6 Å-resolution three-dimensional structure of hCTR1 using electron crystallography, revealing that hCTR1 is a homotrimer with the following key characteristics [30,31]:

(1) 60 amino acids in the extracellular N-terminal domain
(2) three transmembrane (TM) helices (TM1, 2, and 3)
(3) an intracellular loop of 46 amino acids connecting TM1 and TM2
(4) a short intracellular C-terminal domain of 15 amino acids

Later biophysical and biochemical investigations, particularly those using Electron Paramagnetic Resonance (EPR) spectroscopy, elucidated the mechanism of copper translocation by hCTR1 and clarified the specific roles of its C- and N-terminal domains in this process [32,33]. Finally, a high-resolution structure of CTR1 from *Salmo salar* was published, revealing key mechanistic insights of the CTR1 gating mechanism [34]. A detailed discussion of the current structural insights into hCTR1 is presented in the following sections.

In addition to its fundamental function in copper homeostasis, hCTR1 has emerged as a protein of major biomedical and therapeutic interest. Alterations in hCTR1 expression and function have been implicated in cancer, neurodegenerative diseases, and inherited disorders of copper homeostasis [35,36,37]. Furthermore, hCTR1 has attracted attention as a key determinant of cellular uptake of platinum-based chemotherapeutic drugs such as cisplatin (cDDP), linking copper transport to pharmacological responses [19,38,39,40,41,42,43,44,45]. Yeast and mammalian cells lacking CTR1 were resistant to cDDP, while cells overexpressing CTR1 accumulated more drug [39]. Indeed, clinical studies confirmed that hCTR1 expression levels influence sensitivity to platinum-based chemotherapy, particularly in ovarian and lung cancers [38]. Thus, hCTR1 emerged as both a prognostic biomarker and a potential therapeutic target in oncology. Beyond cancer, hCTR1 has been implicated in neurological disorders. Copper imbalance is a hallmark of Alzheimer’s (AD) and Parkinson’s disease (PD), where amyloid-β (Aβ) peptides bind copper and generate oxidative stress [46,47]. Recent studies showed that Aβ peptides can transfer copper to model peptides of hCTR1′s extracellular domain, suggesting a possible functional interaction between amyloids and hCTR1 [48]. This raises the hypothesis that hCTR1 may contribute to synaptic copper handling in neurodegeneration, but in vivo evidence remains limited. Moreover, the human eye, and particularly the retina, is composed of highly specialized neurons [49]. It is therefore not surprising that hCTR1 plays a vital role in maintaining copper homeostasis across ocular tissues [50]. Increased hCTR1 expression has been reported in patients with Eales disease, indicating that dysregulated copper transport may contribute to its pathology [50]. More recently, copper has been identified as a major driver of vision loss in ischemic and diabetic retinopathies through the generation of excessive reactive oxygen species (ROS) [51]. Supporting this, CTR1 knockdown in mice, as well as treatment with copper chelator tetra thiomolybdate, significantly reduced oxidative stress following retinal ischemia–reperfusion injury [51,52]. Current research provides novel implications of hCTR1 in health and disease which highlights the importance of hCTR1 as a hub for copper distribution. Despite three decades of research, crucial aspects of hCTR1 biology remain unresolved, underscoring the need for coordinated, cross-disciplinary studies to fully elucidate its functions in health and disease. The following sections examine the latest insights into the structural and functional properties of hCTR1 and discuss their relevance to human physiology and pathology.

Altogether, these discoveries have advanced our knowledge of hCTR1 from gene identification to a detailed molecular understanding of copper transport and drug interaction. Over the past three decades, converging efforts across genetics, biochemistry, biophysics, pharmacology, and molecular biology have shaped a comprehensive picture of hCTR1′s mechanism, regulation, and roles in health and disease (Figure 2).

## 3. Molecular and Structural Features of hCTR1

### 3.1. Homotrimeric Architecture

**Figure 3:**
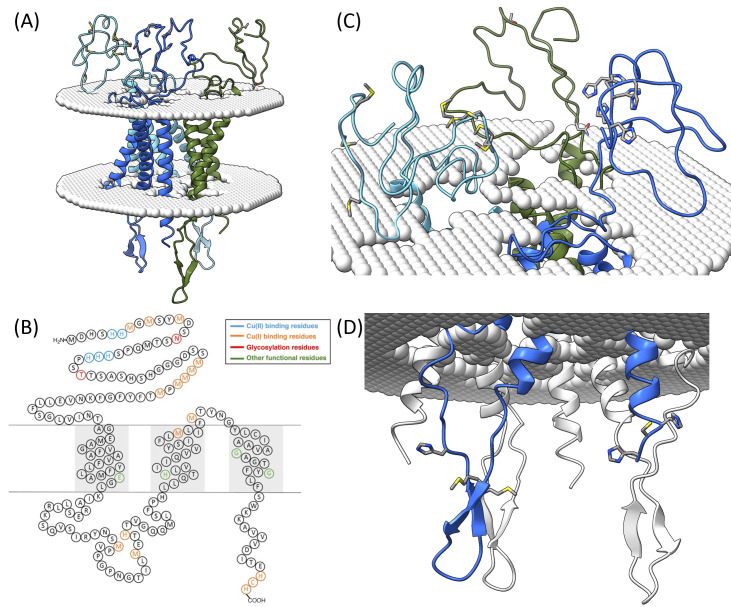
Model of trimeric hCTR1 embedded in a membrane. (**A**) The trimeric hCTR1 model based on PDB-ID: 6M97, created with SWISS-Model, each monomer is represented in a different color. (**B**) hCTR1 sequence in schematic representation highlighting the key residues adapted from [1]. (**C**) N-terminal domain of hCTR1. In light blue, the methionine residues are depicted. In dark blue the histidine residues and in green the glycosylation sites ASP19 and THR27. (**D**) C-terminal domain with relevant key residues involved in copper binding, including the C-terminal ^188^HCH motif depicted in sticks representation.

Biochemical and structural evidence show that hCTR1 functions as a homotrimer. Initial projection structures using 2D electron crystallography revealed a homotrimeric, channel-like assembly with dimensions compatible with ion passage [30]. Crosslinking experiments and biochemical assays confirmed that hCTR1 subunits oligomerize through their transmembrane regions [10,53]. The breakthrough came with the high-resolution X-ray structure of *Salmo salar* CTR1 at ~3 Å resolution, a fusion protein of CTR1 and cytochrome b562 (Figure 3A) [34]. This study revealed threefold symmetry and clearly resolved the methionine triads lining the pore. These methionine rings create a “selectivity filter” for Cu(I), offering soft sulfur ligands that exclude harder ions such as Na(I) or Ca(II) [54]. At the extracellular face, the N-termini extends outward, consistent with biochemical evidence of flexibility and self-interaction (Figure 3C) [33,53,54,55]. Importantly, the homotrimeric assembly is conserved across species, from plants, yeast to mammals [1,34,56]. Structural motifs such as the methionine-rich pore, the glycosylated N-terminus, and the short cytosolic tail are retained, underscoring evolutionary pressure to maintain this architecture (Figure 3B,D).

### 3.2. Domain Organization

The structure of hCTR1 can be divided into three parts with each having a distinct function in copper trafficking: the extracellular N-terminal domain (Figure 3C), the transmembrane (TM) domain (Figure 3A,B), and the intracellular C-terminal tail (Figure 3D).

**The extracellular domain of hCTR1** (Figure 3C) holds two roles. The first one is to capture Cu(II) from the blood carrier protein Human serum albumin [57,58,59,60]. The second one is to reduce Cu(II) to Cu(I) state. The latter remains less clear and based on the connection between iron and copper metabolism; it has been suggested that iron enzymes participate in this reduction [61,62]. In yeast *S. cervisiae* it was suggested that Cu(II) is reduced in the plasma membrane by Fre1 or Fre2 [61,62] enzymes and can then be oxidized from Cu(I) to Cu(II) by Fet3 metalloxidase [63]. In mammals, the situation is more complex, while Dcytb and Steap proteins were proposed to play a role as a cupric reductase [64,65,66]. Additional evidence for enzymatic Cu(II) reduction at the mammalian cell surface comes from liver plasma membrane vesicles, where a membrane-bound NADH oxidase supplies electrons to reduce Cu(II)–histidine or Cu(II)–albumin complexes to Cu(I), thereby stimulating ^64^Cu uptake [67]. In addition, small molecular weight molecules such as ascorbate have been found to mimic this effect, consistent with the required reductive step prior to Cu transport [27]. In human K562 cells, pharmacological ascorbate markedly enhances the release of copper from ceruloplasmin and promotes its accumulation in cells, implying that ascorbate-driven reduction of protein-bound Cu(II) facilitates cellular copper acquisition [68]. Here, ceruloplasmin is transferring the labile Type 1 copper from domain 6 to hCTR1 [69,70,71,72]. Taken together, these studies suggest that mammalian cells rely on iron-dependent surface reductases (Dcytb, STEAPs, plasma-membrane NADH oxidases) and low-molecular-weight reductants such as ascorbate, to ensure that incoming Cu(II) is reduced to Cu(I) before it traverses the hCTR1 pore. Additionally, it was found that the His-Met clusters located in the extracellular loops are crucial in reducing Cu(II) to Cu(I) (Figure 3C).

Owing to difficulties studying the full hCTR1 protein, the first studies focused on the first 14 amino acids, ^1^MDHSHHMGMSYMDS^14^, of the N-terminal domain [73]. Moreover, it was found that two His-Met-Asp motifs of the hCTR1 N-terminus are essential in binding and conserving the oxidation state of copper. It is thus essential that the extracellular domain of hCTR1 holds both Cu(II) and Cu(I) binding sites [60]. Haas and colleagues used spectroscopic experiments to study Cu(I) binding in the extracellular domain. They proposed that Cu(I) binds via N_2_OS coordination to the bis-His sequence, His5 and His6, and to a methionine residue [74]. The group also suggested that the first Cu(II) site involves His3 [74]. In addition, they proposed using UV-VIS and calorimetric titration experiments that the ATCUN (amino-terminal copper and nickel) motif and the bis-His sequence might be two different Cu(II) sites, and that Cu(I) prefers to bind to Met-rich motifs rather than to His-rich motifs. Using spectroscopic and mutagenesis studies on full hCTR1 protein, the Ruthstein laboratory and others have succeeded in showing that the two His-rich sites in the extracellular domain of hCTR1, ^1^MDHxHH and ^22^HHH, serve as two Cu(II) binding sites [75]. Moreover, the Met-rich motifs in the extracellular domain involve two segments, ^7^MxMxxM and ^41^MMMxM, which comprise the first Cu(I) binding sites in the extracellular domain of hCTR1. These segments bind Cu(I) with µM affinity [76,77,78].

The extracellular hCTR1 domain is also characterized by two glycosylation sites, N15 and T27, although N15 is not required for function [79]. Conversely, when O-glycosylation at T27 is prevented, the first 30 amino acids of hCTR1 are cleaved [80,81]. The role of these two glycosylation sites is still not fully resolved. Recent MD work on the hCTR1 N-terminal domain suggested that there is not significant impact of the glycosylation on the conformation of the extracellular domain of hCTR1 [82].

**Figure 4:**
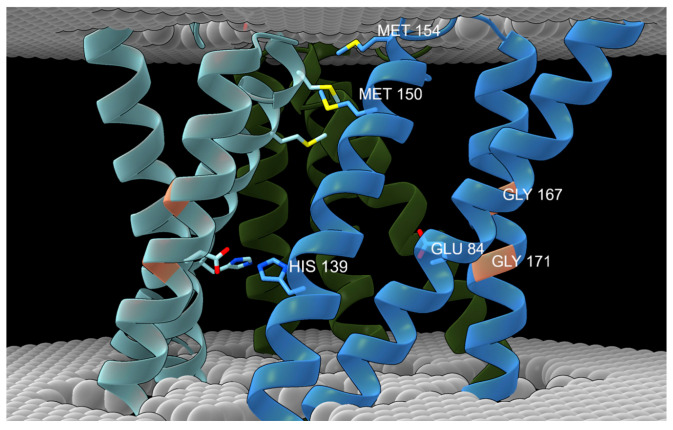
Transmembrane domain of hCTR1 colored by chain. Residues involved in the copper transport are depicted in stick representations, while the conserved residues Gly167 and Gly171 are colored orange for chain A and chain B of the homo trimer. The proximity between the individual monomers promotes the notion that copper transport is carried out between the three monomers and not through a single monomer. Furthermore, the Met residues shown in all three monomers serve as selectivity filter for metal ion transport.

**The transmembrane domain of hCTR1** (Figure 3A,B): Each hCTR1 monomer comprises three TM helices, which are characterized by conserved ^150^MxxxM and ^167^GxxxG motifs. De Feo and co-workers identified TM2 M150 and M154 as Cu(I)-binding residues, whereas TM3 G167 and G171 were proposed to mediate a tight interface between TM1 and TM3 (Figure 4) [31]. Schushan and co-workers constructed a Cα-trace model of the TM domain of hCTR1, which was in close agreement with the experimental structure [83]. Using Gaussian network and anisotropic network models, they proposed functional movements of the TM helices. Based on these movements, the authors proposed a transport mechanism in which Cu(I) ions are transferred one at a time, and M154, along with the conserved residues H139 and E84, control transporter motion as a function of metal ion-binding and pH shift [1]. This model is supported by biochemical experiments showing that H139 and E84 (Figure 4) are indeed important functional residues [28,29]. Later, Tsigelny et al. relied on all-atom simulations to develop a model for the complete hCTR1 structure [84]. Using a Cα-trace approach, these authors suggested that M43, M45, M150, and M154, as well as the ^188^HCH motif in the C-terminal domain (Figure 3D), are important for Cu(I) binding. The importance of M150 and M154 residues to Cu transport is supported by the reported 3 Å-resolution crystal structure of CTR1 lacking the extracellular domain in the apo and holo form and using computational analysis (Figure 3) [34,54]. Moreover, the RCSB PDB database lists five models associated with CTR1 which is the chimera structure CTR1 of *Salmo salar* (PDB-ID 6M98 and 6M97) [34] and three transmembrane NMR structures (PDB-ID: 2LS2, 2LS3, 2LS4) which are pending a literature publication.

**The hCTR1 C-terminal domain** (Figure 3D) has been implicated in copper trafficking and regulation. The C-terminal domain of hCTR1 is composed of 15 amino acid residues (^176^SWKKAVVVDITEHCH^190^). Studies have shown that mutation of Cys^189^ in the hCTR1 C-termini does not affect the Cu(I) transport into the cell, but it might affect copper regulation and transfer to the intracellular copper chaperone [27,28,79,85]. Other studies suggest that this cysteine residue is important for cDDP coordination [86], and for the interaction with a general methionine segment commonly found in metallochaperones [87]. By employing NMR spectroscopy, it was possible to demonstrate that the C-terminus of hCTR1 is directly interacting with the Atox1 metallochaperone but only in presence of Cu(I). If copper was absent, this interaction would be abolished [88]. Further studies confirmed that the C-terminal ^188^HCH motif of hCTR1 is essential for Cu(I) ion transfer from hCTR1 to Atox1 and affects the conformation of Atox1 [32,77,89,90,91]. The Ruthstein group further showed that the intracellular loop between TM1 and TM2 (Figure 3B) can also transfer Cu(I) to Atox1 metallochaperone through a segment which involves Met and His residues [77]. The mechanism and role of Atox1 in the copper transfer was further characterized by EPR spectroscopy and molecular dynamic simulations [75,90,91].

Taken together, the hCTR1 consists of three domains with each holding a dedicated role in copper transport and distribution within the cell. The N-termini coordinates Cu(II) or Cu(I), reduces Cu(II), and preserves the reduced Cu(I) oxidation state. The transmembrane domain acts as a selectivity filter for Cu(I), and the C-terminus controls the distribution of Cu(I). Therefore, hCTR1 can be considered a hub for copper ions, where hCTR1 is warehousing, sorting, and transshipping copper in the human cell.

### 3.3. Gating and Transport Mechanism

The mechanism of copper translocation through hCTR1 is increasingly recognized as a dynamic, gated process, rather than a passive diffusion event. Currently, to our knowledge, no atomic-resolution structure of full-length hCTR1 in a well-defined open, conducting conformation is available. The apo and holo crystal structures of hCTR1 show virtually identical TM1-TM3 backbones, with Cu(I) binding primarily occupying the methionine-based selectivity filter and increasing disorder in the distal C-terminal segment, but without large rearrangements of the TM bundle [34]. Although several computational models, including a Cα-trace elastic-network analysis of the TM bundle and an all-atom hCTR1 model, have suggested how methionine-rich motifs and the flexible C-terminus might gate Cu(I) passage, none of these studies has yet yielded an experimentally determined, atomic-resolution structure of full-length hCTR1 captured in a clearly conducting state [83,84]. Recent MD work on full-length hCTR1 has begun to connect Cu(I) binding at the intrinsically disordered N-terminus to opening of the transmembrane pore. Aupič et al. showed that Cu(I) ions and the membrane environment promote compaction and α-helical structure in the N-terminal ectodomain, allowing Met1 to insert into the lumen and engage the top selectivity-filter with Met154 [92]. This interaction drives a lipid-assisted conformational switch of TM2 from a bent-close around residues Val143-Tyr147/Phe148 to a more linear-open state, which propagates along a defined signal-transduction pathway to the TM2/TM3 interface and the cytosolic end of TM3, where partial unfolding displaces the C-terminal gate from the pore. Complementary QM/MM metadynamics on the *S. salar* TM domain demonstrate that Cu(I) occupancy at the two methionine triads strongly biases the rotameric states of their methionine side chains and thereby controls water entry and Cu(I) release toward the cytosol, underscoring the key role of selectivity-filter conformational plasticity in gating [54].

Recently, computational and spectroscopic measurements revealed conformational flexibility and movements detailing the transport mechanism by hCTR1 [1,55,75,92,93,94]. Using distance EPR measurements in vitro and *in-cell*, the Ruthstein group has succeeded in shedding light on the role of the extracellular and intracellular domain of hCTR1 in copper trafficking. The group showed that at specific copper concentration, the extracellular domains are approaching the lumen while simultaneously, the C-terminal tails penetrate the lumen to collect the Cu(I) ions from the pore [75,94]. In addition, hCTR1 was shown to coordinate two Cu(II) ions in each monomer and up to five Cu(I) ions [75]. This capacity suggests that the N-terminal region acts as both a sensing gate and a copper repository, stabilizing multiple oxidation states before transferring Cu(I) to the methionine filter in the pore. Such buffering could be especially relevant in synaptic or endosomal environments where copper fluctuates between redox states. Together, these findings support a facilitated diffusion model in which copper ions sequentially bind to histidine and methionine motifs in the extracellular domain, while conformational changes in both the extracellular, transmembrane domain and the intracellular domains of hCTR1 regulate copper transfer. Although it remains unclear whether this process depends on auxiliary cofactors or cytosolic chaperones, growing evidence indicates that hCTR1 operates as a dynamic, adaptable transporter rather than a passive channel, delicately balancing the flexibility, and selectivity of copper ions.

## 4. Regulation of hCTR1

### 4.1. Copper-Dependent Regulation

**Figure 5:**
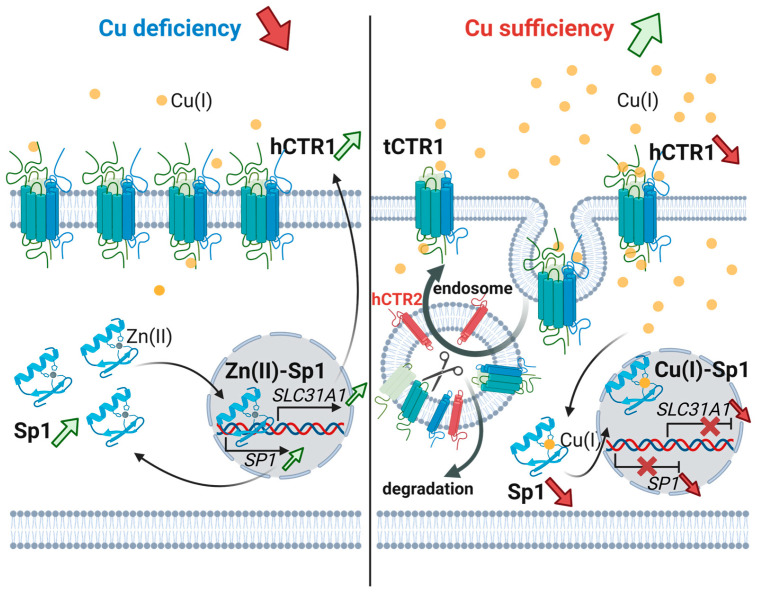
Copper-dependent regulation of hCTR1 abundance and activity. The schematic illustrates the coordinated post-translational and transcriptional mechanisms that regulate hCTR1 in response to cellular copper levels in conjunction with zinc. Left, Cu deficiency: Low extracellular Cu(I) promotes stable homotrimeric hCTR1 at the plasma membrane, enabling efficient copper uptake. Under these conditions, the transcription factor Sp1 binds zinc in its zinc-finger motifs (Sp1–Zn), allowing it to associate with the *SLC31A1* and *SP1* promoter and upregulate hCTR1 and Sp1 expression. Right, Cu sufficiency: Elevated Cu(I) triggers rapid post-translational regulation. Excess copper induces proteolytic cleavage of the extracellular Met-rich domain and stimulates endocytosis of hCTR1 into endosomal compartments, thereby reducing copper influx. Subsequently, the truncated hCTR1 (tCTR1) is eventually recycled to the plasma membrane. Simultaneously, copper binding to Sp1 (Sp1–Cu) disrupts its DNA-binding ability, leading to transcriptional downregulation of *SLC31A1* and *SP1*. The combined action of (i) copper-dependent hCTR1 internalization and recycling and (ii) suppression of *SLC31A1* and *SP1* transcription maintains copper homeostasis and prevents toxic copper overload. Transcriptional regulation of hCTR1 via *EPAS1* is omitted in this figure for simplicity.

hCTR1 is the principal high-affinity Cu(I) importer in human cells. However, as mentioned earlier, copper overload promotes reactive oxygen species (ROS) generation via Fenton chemistry, resulting in oxidative damage and cellular toxicity [95,96,97]. Consequently, cells maintain strict translational and post-translational control of hCTR1 to prevent copper overload [98,99]. A key mechanism is copper-stimulated endocytosis of hCTR1. Within minutes of exposure to elevated copper as low as 2 µM, hCTR1 is rapidly internalized from the plasma membrane (Figure 5) [99]. Another proposed mechanism suggests that excess copper triggers monomerization of homotrimeric hCTR1, thereby preventing further copper influx [100]. Interestingly, it was previously shown that protein oligomerization regulates the activity of yet another copper-sensing protein in prokaryotes [101]. This parallelism implies that modulation of protein function through oligomerization is an evolutionarily conserved regulatory strategy across a diverse range of organisms [102,103]. Additionally, regulatory endocytosis of hCTR1 was shown to be clathrin dependent [99]. The internalized hCTR1 is found in early endosomes, indicating a canonical clathrin-mediated endocytic route. Importantly, copper-triggered endocytosis of hCTR1 is reversible (Figure 5). When extracellular Cu is depleted or reduced, internalized hCTR1 is re-routed to restore copper uptake capacity [104]. These dynamic trafficking events together with the regulatory oligomerization state of hCTR1 enable acute regulation of copper entry as a safeguard against toxicity. Notably, prolonged high copper concentration can also target internalized hCTR1 for lysosomal degradation, leading to a net decrease in hCTR1 protein levels. This interplay between rapid retrieval and downregulation constitutes a potent post-translational and translational feedback loop controlling copper influx [99].

At the transcriptional level, the mammalian *SLC31A1* gene is regulated by the transcription factor Sp1 (Figure 5). Sp1 is a transcription regulator with zinc finger domains in which Zn(II) can be displaced by Cu(I), thereby inhibiting its DNA-binding capacity and downregulating *SLC31A1* transcription [45,85,105,106,107,108]. Sp1 is not only the Cu-sensing activator of *SLC31A1*, but also auto-regulates its own transcription [109,110] (Figure 5). Copper-dependent changes in Sp1 DNA binding simultaneously tune both *SLC31A1* and SP1 transcription, creating an oscillatory feedback loop that stabilizes cellular copper homeostasis [110]. Other studies have shown that *EPAS1* (*HIF2α*) regulates the basal transcription of *SLC31A1* in an oxygen sensitive manner [111]. In addition, extreme copper deficiency or overload can exert some effects on hCTR1 levels. For example, in mice, perinatal copper deficiency led to increased hCTR1 protein in certain organs, suggesting a compensatory upregulation [112].

Altogether, hCTR1 abundance is regulated on both the protein and transcriptional levels (Figure 5). Post-translational regulation provides the most immediate response to fluctuations in copper availability, enabling rapid internalization and recycling of hCTR1 and thus efficiently preventing copper overload [99]. At the transcriptional level, hCTR1 (*SLC31A1*) expression is controlled primarily by two transcription factors: Sp1, which is metal sensitive, and *EPAS1* (*HIF2α*), which regulates *SLC31A1* transcription in an oxygen-dependent manner. Through the combination of fast trafficking-based control and basal transcriptional regulation, cells maintain intracellular copper within a narrow and safe range while ensuring adequate supply for essential cuproenzymes.

### 4.2. Proteolytic Cleavage

Interestingly, hCTR1 is controlled via a third, to our knowledge unique regulation mechanism for transmembrane metal transport proteins [81,113]. The group of J. H. Kaplan discovered and characterized this critical regulatory mechanism as being proteolytic cleavage of hCTR1′s extracellular domain in response to elevated copper (Figure 5) [81]. Mutagenesis studies revealed that lack of Thr^27^ glycosylation results in a proteolytic cleaved transporter residing in the plasma membrane with around 55% of its transport capacity [81]. In-cell experiments revealed the biological implication of this truncation. Upon exposure to elevated copper, a fraction of hCTR1 that has been endocytosed undergoes proteolytic processing in endo-lysosomal compartments. Specifically, the long N-terminal extracellular domain of hCTR1, which contains the His-Met-rich metal-binding motifs, is cleaved off by endo lysosomal cathepsin proteases [114]. Whether the N-terminal fragment released by proteolysis is exclusively cleaved in a holo-(Cu-bound) or apo- (metal-free) state remains not fully understood. The cleavage of the holo-state is an attractive model. Here, lysosomal excretion of a Cu-loaded N-terminal peptide would directly reduce intracellular copper burden, and the released copper would not be available for import. It was shown in vitro that peptides capable of chelating metals such as Zn or Cu are more stable in their holo state compared to their apo configuration [115]. Here, the coordination of metal prevents or reduces hydrolytic degradation and increases the peptides’ stability [115]. More specifically, experiments on the hCTR1 N-terminal region showed that at lower pH reduced Cu(I) remains bound to the peptide unlike Cu(II) [116]. This data further supports an intact excretion of the Cu(I) bound N-terminal fragment. Moreover, it was shown in the past that specific peptides can be spared and are excreted through the lysosomal pathway [117]. However, direct evidence for the excretion of the truncated N-terminal hCTR1 holo-peptide is still missing.

Nevertheless, this model is speculative and needs direct biochemical and cell biological validation. Overall, this cleavage yields a lower molecular weight truncated hCTR1 that remains membrane-associated but lacks the copper binding N-terminus. Functionally, the N-terminal domain is needed for maximal copper uptake, therefore its removal reduces copper import capacity compared to full-length hCTR1 [80]. Cleavage is therefore a means to post-translationally inactivate a portion of hCTR1 transporters when copper is abundant. Importantly, this process is reversible on a population level. New hCTR1 can be synthesized and delivered to the membrane over time, and truncated hCTR1 may still fulfill a key role in copper distribution.

The truncated hCTR1 has a comparable size to the hCTR2 protein. hCTR2 shares a surprisingly low sequence identity of ~40% amino acid with hCTR1 and is predicted by AlphaFold models and experiments to adopt a similar trimeric three-transmembrane architecture [118]. Moreover, the amino acid residues that are essential for hCTR1 activity are conserved in hCTR2 and are also required for mobilizing Cu from the vacuole [119]. Still, hCTR2 is considerably shorter and lacks both the long His/Met-rich N-terminal ectodomain and the C-terminal HCH motif that define hCTR1, indicating that the two transporters are not simply redundant copies of one another [120,121]. The structural differences indicate that hCTR1 and hCTR2 occupy distinct cellular niches and operate in complementary copper transport regimes, hCTR1 resides predominantly at the plasma membrane, where it mediates high-affinity Cu(I) uptake from the extracellular milieu and is essential for systemic copper acquisition, whereas hCTR2 localizes mainly to late endosomes and lysosomes, with only a minor amount detectable at the cell surface, consistent with a role in mobilizing copper from acidic intracellular pools rather than serving as the major entry route [120,122]. Studies have shown that the biogenesis of truncated hCTR1 requires CTR2 (Figure 5) [123]. In cells or mice lacking CTR2, hCTR1 fails to undergo normal extracellular cleavage [123]. Consequently, those CTR2-knockout mice accumulate excess copper in intracellular vesicles and have elevated tissue Cu levels. This indicates CTR2 facilitates either the cleavage or stabilization of truncated hCTR1, forming a key part of the regulatory circuit.

Full-length hCTR1 is essential for initial copper uptake at the plasma membrane, but the truncated form, without the high-affinity binding domain, is needed to release or redistribute copper from internal compartments. Thus, when copper is plentiful, the cell shifts hCTR1 from an “import mode” to a partially inactive, truncated state that can still transfer copper out of endosomes to the cytosol for utilization but reduces any further influx from outside. This sophisticated mechanism prevents excess influx while not wasting the copper that has already been taken up.

Proteolytic down-regulation of hCTR1 affects not only physiological copper homeostasis but also the transport of other metals and metallodrugs. The extracellular Met-rich domain that is cleaved is essential for the uptake of other compounds. For example, platinum-based anticancer drugs such as cDDP rely, at least in part, on hCTR1 as an entry route into cells, meaning that copper or cDDP induced cleavage of this domain can contribute to chemoresistance [19,124]. The presence of the Met-rich N-terminus is required for efficient platinum drug uptake [19]. High copper or cDDP exposure can induce N-terminal hCTR1 cleavage, thereby reducing cDDP uptake [114]. This mechanism is still controversial but might be one factor of how tumor cells acquire cDDP resistance [125]. More detailed aspects of the controversial role of hCTR1 in cancer are discussed below.

In summary, proteolytic cleavage of hCTR1 serves as a copper-dependent “off switch” that blunts further uptake and teams with CTR2 to safely sequester and later redistribute copper in sub-cellular compartments, all while potentially modulating the cellular distribution of platinum drugs.

### 4.3. Crosstalk with Other Metals

Although hCTR1 is highly selective for copper due to the rich methionine transmembrane selectivity filter, copper homeostasis does not operate in isolation. There is a substantial crosstalk between copper and other metals at the level of transport and regulation. One documented but still controversial direct interaction is the one with zinc. Zn(II) is known to antagonize copper uptake in vivo, high zinc can induce a functional copper deficiency [126,127,128]. In fact, oral zinc therapy is used to treat Wilson’s disease patients by blocking intestinal copper absorption [129,130]. Part of this effect is indirect, because zinc upregulates metallothioneins that bind copper [126,131]. However, there might also be a direct interplay with hCTR1. The chimeric protein structure of *Salmo salar* CTR1 revealed an allosteric zinc-binding site involving E84 and H139R [34]. It appears that when Zn(II) occupies this allosteric site, it can attenuate hCTR1 activity and promote an endocytosis-like conformational change. The same study showed that hCTR1 mutants that disrupt the putative Zn site (H139R or E84Q) exhibit increased basal copper uptake and diminished copper-induced internalization [34]. Thus, there might be a possibility that Zn(II) acts as a direct feedback modifier of hCTR1. At elevated concentrations, zinc can bind to hCTR1 and thereby moderate copper transport, adding an additional layer of homeostatic control. However, the Zn(II) concentrations required to produce this effect in vitro are far above physiological levels. As a result, physiological relevance still needs to be demonstrated [36]. Additionally, earlier studies were unable to reproduce these findings. In contrast, the addition of Zn(II) or Cd(II) was reported not to significantly interfere with copper uptake [28]. Nonetheless, the concept of Zn-Cu antagonism through an allosteric binding site of hCTR1 is intriguing and represents a direct metal–metal regulatory interaction [132]. But more research is needed to support the potential allosteric modulation of hCTR1 by zinc.

Given the role of zinc in the transcription regulation of hCTR1 it is counterintuitive that zinc regimen would result in reduced copper uptake. Despite being counterintuitive, zinc might induce lower copper uptake albeit through a different mechanism. Here, metal regulatory transcription factor 1 (MTF1) offers an additional layer of explanation for the clinical use of zinc salts in conditions of copper overload such as Wilson’s disease [128,133,134]. Pharmacological doses of Zn(II) increase the abundance of intestinal and hepatic metallothionein regulated by MTF1, thereby sequestering incoming Cu(I) and preventing its transfer into the circulation [133,135,136]. Thus, the antagonistic effect of Zn(II) on copper uptake is not mediated through the transcription factor Sp1. Instead, zinc acts mainly by stimulating MTF1 driven metallothionein expression in enterocytes, which sequesters dietary copper and prevents its absorption in the gastrointestinal tract.

Altogether, the interaction between zinc and copper is highly complex. For clarity, we propose four principal levels of crosstalk. The first occurs at the level of intestinal absorption. The second involves transcriptional regulation of *SLC31A1*. The third reflects direct molecular interactions with hCTR1. The fourth arises from zinc-dependent modulation mediated by Zinc Transporter 1 (ZnT1). The first and most clearly defined level of interaction occurs at the stage of intestinal absorption. Excess zinc induces the expression of metallothioneins, which tightly bind and sequester copper, thereby reducing its bioavailability [133,135,136]. The second level involves transcriptional regulation of *SLC31A1*, where the binding of zinc or copper to the transcription factor Sp1 leads to upregulation or downregulation of hCTR1 expression, respectively [45,85,105,106,107,108]. The third level of interaction is more controversial and remains inconclusive in the literature. A direct interaction between zinc and hCTR1 via an allosteric site has been reported in a chimeric crystal structure from *Salmo salar* [34]. More evidence for zinc-dependent fine-tuning of hCTR1 comes from a comprehensive study showing that a 10- or 50-fold excess of zinc indeed reduces copper uptake [27]. The same work demonstrated a pH dependence of transport and highlighted the impact of experimental variables such as expression system and cell line choice, which likely contribute to conflicting findings across studies. Indeed, others report no effect of zinc supplementation in HEK293 or Caco-2 cells [28]. Interestingly, knockdown experiments revealed that hCTR1 itself can import zinc and iron, suggesting that the transporter may participate more broadly in cellular metal homeostasis than previously assumed [137]. Therefore, the proposed interaction or competition between zinc and copper at the level of hCTR1 requires further investigation. It remains unclear whether zinc-dependent inhibition of copper uptake is cell-type specific, reflects true competitive binding at hCTR1, or arises from experimental artifacts. The fourth level of crosstalk involves direct competition between copper and zinc at ZnT1, which mediates the import of both Zn(II) and Cu(II) ions [138]. Because ZnT1 can transport copper independently of hCTR1, this provides an alternative entry route for copper into the cell. Such parallel import pathways may help explain the complexity seen at the third level of crosstalk and why elevated zinc concentrations can reduce apparent copper uptake in certain experimental systems.

Another important crosstalk is silver. Ag(I) is chemically similar to Cu(I), both are monovalent “soft” cations, and indeed silver can enter cells via hCTR1 [139,140,141,142]. Experimentally, silver ions have been shown to inhibit copper uptake in cultured cells by blocking hCTR1 [143,144]. Biochemical studies have shown that Met43 and Met45 within the N-terminal domain are key residues responsible for Ag(I) coordination [78,145]. These experiments also revealed that interactions of the N-terminus with the adjacent membrane surface significantly influenced Ag(I) binding affinity. Comparable observations were made for Cu(I), as demonstrated by EPR analyses showing that the N-terminal domain undergoes distinct structural rearrangements depending on its adjacent lipid environment [94]. Together, these findings strengthen the idea that hCTR1 employs an environment-dependent gating mechanism during metal binding and transport.

Potentially, the presence of non-physiological metals like silver triggers a copper-like response. Cells exposed to Ag(I) may interpret it as a signal of copper excess, potentially triggering hCTR1 internalization or degradation as a protective response to prevent inappropriate metal influx. However, this remains a tentative hypothesis, as experimental data on the interaction between silver and hCTR1 are limited. The ability of Ag(I) to hijack hCTR1 underscores the transporter’s preference for soft Lewis acids and shows that copper uptake can be perturbed by other group 11 metals. This cross-reactivity has practical implications: for instance, silver-based antimicrobials could disrupt human copper homeostasis by competing for hCTR1, and dietary silver, or even high copper concentrations itself, could reciprocally interfere with zinc and iron metabolism due to shared regulatory networks.

Copper homeostasis also intersects with platinum, which is very well documented in the context of chemotherapy. As noted, the platinum drug cDDP enters cells partly via hCTR1. This creates a form of “crosstalk” between copper and platinum. Cells modulate hCTR1′s abundance, activity, and expression in response to copper availability (Figure 5); this regulation will consequently alter cDDP uptake. Moreover, cDDP triggers the same regulation mechanism as copper (Figure 5), through Sp1 and the above-described degradation or recycling mechanism [38,109,127]. For example, raising extracellular copper can reduce cDDP uptake and toxicity, likely by downregulating hCTR1 at the membrane [109,146]. Conversely, copper chelation or deficiency might increase hCTR1 levels and potentially enhance cDDP import, although the relationship is complex [109,124,146]. Studies have found a significant correlation between cellular hCTR1 levels/localization and cDDP accumulation and sensitivity to the drug [114,124,147]. Tumor cells often exploit this by increasing degradation of hCTR1 in endosomes when exposed to cDDP, thereby becoming drug-resistant while simultaneously exhibiting signs of perturbed copper homeostasis [148]. These examples underscore a broader principle wherein many transition metals and metal-containing compounds modulate each other’s transport pathways and regulatory mechanisms. Consequently, a full understanding of the complex role of hCTR1 requires a comprehensive perspective that encompasses all ligands, whether allosteric or competitive.

### 4.4. Interaction with Chaperones

Once copper ions cross the plasma membrane via hCTR1, they are immediately intercepted by a network of intracellular copper chaperones [1]. This handoff is vital because unbound copper can catalyze production of ROS. As a result, free Cu(I) in the cytosol is almost undetectable and estimated only at femtomolar levels [149,150]. Cu(I) ions are transferred from hCTR1 to specific chaperone proteins that safely escort copper to its destinations in the cell. The key copper chaperones in human cells include Copper transport protein (Atox1), Copper chaperone for superoxide dismutase (CCS), and Cytochrome c oxidase copper chaperone (COX17) (Figure 1). Atox1 is a soluble cytosolic protein that delivers copper to the secretory pathway P-type ATPases, more specifically ATP7A which is found in cell and trans-Golgi network membranes, while ATP7B is mainly found in the trans-Golgi network membranes [151,152]. Subsequently, these cation transport ATPases export excess copper. The second chaperone, CCS acquires copper for the maturation of cytosolic superoxide dismutase. And lastly, COX17 and related mitochondrial chaperones transfer copper to the mitochondria for cytochrome c oxidase assembly.

To date, most of the focus has been on the Atox1 copper chaperone and the copper transport ATPases ATP7A/B [153,154,155,156,157]. The crystal structures of Atox1 [158], and its analog yeast Atx1 were reported at a 1.02 Å resolution in both holo and apo state [159,160,161]. Atox1 was shown to be a small soluble protein of 68 amino acids, with an overall βαββαβ fold structure. It coordinates one copper atom via cysteine residues in a conserved MxCxxC motif [156,162,163]. Also, this data was supported by solution NMR spectroscopy [164]. Moreover, it was shown that Atox1 binds to cisplatin and oxaliplatin at the same site in a dimeric manner, albeit to a lesser extent than copper [165]. The ATP7A/B copper pumps contain between two and six MxCxxC metal-binding domains (MBDs) at their amino terminal, each with an overall tertiary structure similar to the one of Atox1, where each MBD binds a single copper atom in vitro. According to structural predictions, the metal is transferred via a series of ligand-exchange reactions involving two- and three-coordinate intermediates between cysteine ligands in the CxxC motifs on Atox1 and the recipient copper-transporting ATPases [153,156,157,162,166,167]. A recent publication reported the cryo-EM structure of ATP7B interacting with Atox1, revealing a detailed transport mechanism of ATP7B and the interaction with Atox1 [168]. Others found via crystallographic experiments that despite the presence of EDTA, Zn remained coordinated in Atox1 [169]. Thus, it led to the conclusion that the two metabolisms between Zn and Cu are intertwined in human cells even on the Atox1 chaperone level [169]. This interaction further supports the alleviating effects of Zn regimen in Wilsons disease as described above.

hCTR1′s structure and sequence are well-suited for coordinating with Atox1 chaperone. Each hCTR1 subunit has a cytosolic C-terminal tail ending in a conserved HCH motif (Figure 3D), that has been shown to bind Cu(I) and is directly involved in passing copper to Atox1. The Ruthstein group has shown using various biophysical methods that the C-terminal domain of hCTR1 can hold a copper ion and transfer it to Atox1 [32,89]. In this study, deletion of the C-terminal 3 amino acids (removing the HCH) abolished copper delivery to Atox1, underscoring its role as the handoff site [32]. Moreover, structural measurements suggest that the cytosolic tail might undergo copper-dependent conformational changes, positioning the HCH motif to deliver the metal to Atox1 [32,89]. Later, Kahara et al. showed that these last three amino acid residues of the Cu(I) binding site, and mutations in this motif decreased the binding of Cu(I) but did not eliminate it [88]. The intracellular loop between TM1 and TM2 of hCTR1 also contributes to this relay. It holds a His and two Met residues that can coordinate Cu(I) and was found to closely interact with Atox1 [77]. Thus, as a Cu(I) ion exits the hCTR1 pore into the cytosol, it is likely captured transiently by either the internal loop or the C-terminal tail of hCTR1, and from there transferred directly to a chaperone by ligand exchange. This proximity-based transfer mechanism ensures that copper ions are not free in the cytosol and move from hCTR1′s binding site to Atox1′s cysteine-rich active site.

Once Atox1 receives the Cu(I), it ferries it through the cytosol to the Trans-Golgi network, and cell membrane docking with ATP7A/7B. These ATPases then export the copper to vesicles or extracellular space where it is inserted into proteins like ceruloplasmin, dopamine-β-hydroxylase, and lysyl oxidase [170,171,172]. Notably, ATP7A and ATP7B themselves are regulated through the interaction with copper bound Atox1 [173,174]. At high copper levels, ATP7A traffics from the Golgi to the cell surface to export excess copper [173]. This means that at high copper conditions, hCTR1 is reduced in the plasma membrane and at the same time ATP7A is moving to the plasma membrane, in a coordinated response that promotes copper efflux over influx [173,175]. More literature about the structure, function, and role in disease involving ATP7A and ATP7B can be found in the following reviews [176,177].

Meanwhile, the CCS-SOD1 branch is now recognized to be more directly coupled to hCTR1 than previously appreciated. CCS possesses a positively charged membrane-binding surface that allows it to encounter Cu(I)-loaded hCTR1 C-termini at the plasma membrane. Structural and biochemical studies in yeast and human systems show that CCS and apo-SOD1 are recruited to the vicinity of hCTR1 where cellular membranes act as scaffolds, lowering the dimensionality of the search problem and focusing Cu(I) delivery to SOD1 [178]. In this context, CCS can form a CCS-hCTR1-SOD1 heterotrimer, in which CCS bridges the cytosolic metal-binding tail of hCTR1 and immature SOD1 and orchestrates Cu(I) insertion and disulfide bond formation. Once SOD1 is fully metalated, CCS disengages from hCTR1, and the ternary complex is terminated [179,180]. These data support a model in which CCS is selectively recruited to Cu(I)-loaded hCTR1 when there is unmet SOD1 demand, rather than simply sampling a homogeneous cytosolic Cu(I) pool.

Importantly, the Atox1 and CCS pathways are not completely insulated from one another. In vitro human Atox1 and the first domain of CCS can exchange Cu(I) directly in both directions, and this cross-reactivity persists with full-length CCS, implying that the secretary Atox1-ATP7A/B and CCS-SOD1 branches can re-distribute Cu(I) between themselves depending on relative demands [181]. Together with the comparable, very high Cu(I) affinities of these chaperones, this suggests that they compete for a common, small “labile” Cu(I) pool that is ultimately supplied by hCTR1 and buffered by glutathione and metallothionein [170,182].

COX17 represents the mitochondrial arm of this network. It is a small cysteine-rich protein that resides in the cytosol and mitochondrial intermembrane space, where it forms polycopper complexes and transfers Cu(I) to SCO1/SCO2 and COX11 for assembly of the Cu(A) and Cu(B) centers of cytochrome c oxidase [183,184,185,186]. NMR and biochemical studies show that Cu(I) binding to COX17 is tightly coupled to its oxidative folding, with specific disulfide patterns acting as redox switches that control Cu(I) loading and interaction with other proteins [185,187]. Although COX17 clearly depends on hCTR1 for its Cu source, there is currently no evidence for a stable COX17-hCTR1 complex analogous to the CCS-hCTR1-SOD1 heterotrimer. Instead, COX17 is thought to acquire Cu(I) from the same glutathione-buffered cytosolic Cu(I) pool that is maintained by hCTR1 and modulated by ATP7A/B, with mitochondrial Cu flux controlled primarily by the abundance and redox state of COX17 and its downstream assembly factors rather than by direct gating at the importer [180,188,189].

Taken together, these observations argue against a model in which hCTR1 either indiscriminately hands Cu(I) to any available chaperone or enforces a rigid hierarchy that prioritizes mitochondria over secretory pathways or vice versa. Instead, hCTR1 appears to create a high-affinity Cu(I)-loaded hub at the cytosolic face of the plasma membrane, from which multiple chaperones are recruited in a context-dependent manner. Atox1 and CCS can both interact directly with the Cu(I)-bound C-terminal tail of hCTR1, with CCS additionally forming a ternary complex with SOD1 that couples its residency at the transporter to the unmetalated SOD1 pool. COX17, by contrast, seems to access Cu(I) mainly via the downstream labile pool, with its mitochondrial targeting and Cu-donor activity governed by cysteine redox state and partnership with SCO1/2 and COX11. Moreover, a network-level analyses of mammalian Cu homeostasis reinforce this view, emphasizing that intracellular Cu fluxes are dynamic and cell-type specific, with secretory, cytosolic and mitochondrial branches all drawing from a shared exchangeable Cu(I) reservoir whose partitioning is tuned by chaperone expression, localization, and redox environment rather than by a single “sorting switch” at hCTR1 [190].

## 5. hCTR1 in Physiology and Pathology

### 5.1. Systemic Copper Homeostasis

hCTR1 controls the main entry points for dietary and circulating copper at the organismal level, acting as the rate-limiting high-affinity importer at barrier epithelia and specialized interfaces [99,191,192]. In mice, whole-body CTR1 knockout is embryonic lethal, establishing an essential role for organismal copper acquisition early in development [25,26]. In adults, hCTR1 is enriched in tissues sensitive to copper and tissues that are in contact with copper. Notably the digestive system, intestine, liver, brain interfaces, testis, placenta, and others. These levels are tissue-specific and responsive to copper levels. From a systems perspective, human dietary copper absorption and distribution are tightly constrained. Classic human balance studies estimate ~1 mg/day net intestinal absorption with extensive endogenous biliary/intestinal fluxes [193,194].

**Figure 6:**
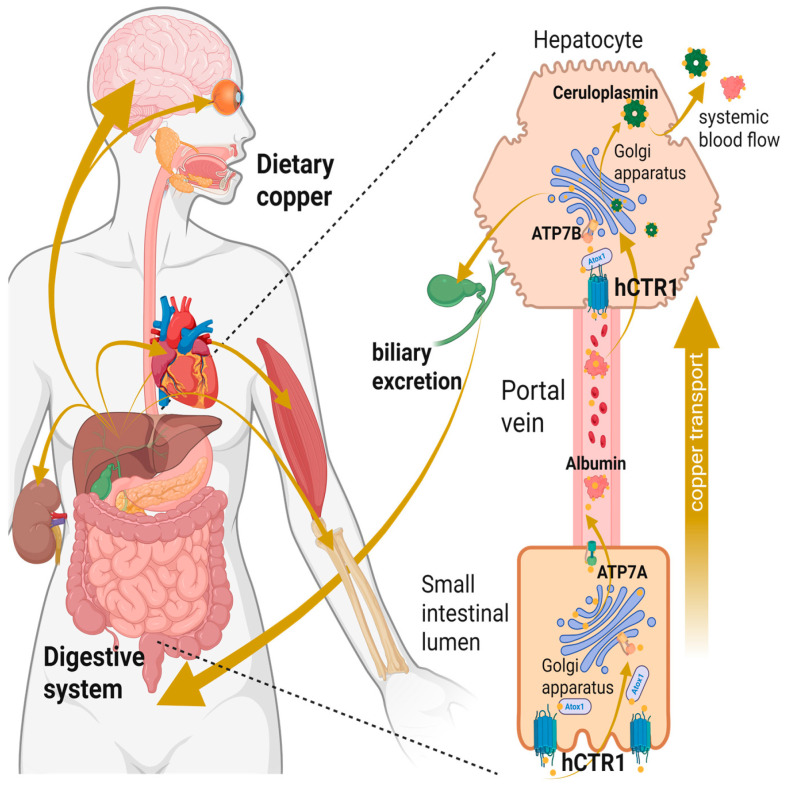
Systemic and Molecular Pathways of Copper Homeostasis. Left**,** overview of copper distribution. Dietary copper is absorbed by the digestive system and transported to the liver via the portal vein. From the liver, copper is distributed to peripheral tissues (brain, heart, muscle, kidney) or excreted via the bile. Right**,** cellular mechanisms of copper transport. Copper enters enterocytes from the intestinal lumen via the transporter hCTR1. Intracellular copper is chaperoned by Atox1 to the Golgi apparatus and exported across the basolateral membrane into the portal vein by ATP7A. In the blood, copper binds to Albumin and travels to the liver. Hepatocytes take up copper via hCTR1. Inside the hepatocyte, ATP7B mediates the incorporation of copper into Ceruloplasmin for systemic release or facilitates biliary excretion.

In the intestine, genetic and localization work shows CTR1 is the apical Cu(I) importer of enterocytes found in the intestinal lining. Therefore, intestinal hCTR1 deletion compromises dietary copper absorption and leads to systemic deficiency [195,196]. This apical positioning explains the clinical observation that luminal factors like dietary Zn(II), Fe(III)/Fe(II), and chelators can modulate copper entry via competition or indirect regulatory networks [197,198]. At the luminal microvilli of the small intestine, soluble copper ions are imported by enterocytes and subsequently released to the blood stream (Figure 6) [196]. Within the bloodstream, copper is loosely bound to albumin and is conveyed to hepatocytes through the portal vein in the liver [57,193,199]. The hepatocytes cells buffer systemic copper and orchestrate whole-body distribution via ATP7B-dependent biliary excretion and cuproprotein synthesis, while hCTR1 sits upstream of this hepatic control determining how much copper arrives for distribution [193].

At barrier tissues beyond gut and liver, hCTR1 also contributes to fetal–maternal and brain copper handling (Figure 6). hCTR1 is expressed in human placenta and trophoblast-derived cell lines, consistent with a role in placental transfer of copper to the fetus [200]. In the central nervous system, hCTR1 mRNA and protein levels are detectable in brain–fluid interfaces including choroid plexus and brain micro vessels [112,201]. Together, these data position hCTR1 as a bottleneck for systemic copper flow, meters uptake at the gut, participates in placental and brain copper delivery, and ensures that hepatocytes receive adequate copper for downstream distribution.

### 5.2. hCTR1 Related Inherited Neurodegenerative Diseases

Copper imbalance is repeatedly implicated across various neurodegenerative disorders, including Alzheimer’s disease (AD), Parkinson’s disease (PD), Huntington’s disease (HD), prion diseases, and Amyotrophic lateral sclerosis (ALS) [46,192,202,203,204]. Regarding hCTR1 specifically, immunohistochemical and transcript studies suggest changes in hCTR1 expression at brain interfaces and within vulnerable brain regions. In AD literature, several reports argue for perturbed copper availability in hippocampus and cortex [35,205,206]. Mechanistically, there is growing interest in how extracellular ligands might supply Cu(II) to the extracellular domain of hCTR1 in the synaptic cleft. Recent biochemical work proposed that Aβ peptides can participate in Cu(II) transfer to hCTR1 [48]. Complementary studies on hCTR1′s N-terminal Cu(II) binding support the idea that serum albumin and other carriers may transiently dock near the hCTR1 extracellular domain to transfer copper, processes that, if disturbed, could impact neuronal copper delivery [57].

Some studies suggest that dietary copper intake may slow the progression of AD [207,208]. Higher brain copper levels have been associated with slower cognitive decline and with reduced AD and PD pathology. Interestingly, a recent meta-analysis reported elevated copper levels in the blood of AD patients but reduced levels in PD patients, while both groups showed decreased copper concentrations in postmortem brain tissue [46]. This apparent mismatch between peripheral and central copper pools supports the hypothesis that copper dyshomeostasis contributes directly to AD and PD pathogenesis [207,209]. Despite these associations, the mechanistic link between dietary copper intake, systemic copper distribution, and regional copper levels in the brain remains unresolved. Current evidence indicates that both copper excess and copper deficiency can promote neurodegeneration, highlighting the narrow therapeutic window required for neuronal copper balance [210,211,212]. This tight homeostatic range makes therapeutic strategies, whether copper chelation or copper supplementation, extremely challenging in AD and PD, as even subtle shifts may worsen disease progression rather than alleviate it. Therefore, targeting hCTR1 directly to improve neurodegenerative conditions such as AD or PD seems rather difficult given the small therapeutic window of copper.

The hCTR1-Atox1-ATP7A/B axis is also associated with Menkes disease and Wilson’s disease [16]. In both conditions, the pathology is driven primarily by mutations in proteins that regulate copper homeostasis, most notably disease-causing mutations in ATP7A or ATP7B, which lead to systemic copper deficiency or copper overload [213,214]. hCTR1 sits upstream of both ATPases. If hCTR1-mediated copper import fails, downstream ATP7A/B function cannot compensate, because cytosolic Cu(I) does not reach [215,216]. Clinically, hints of *SLC31A1* involvement in human disease accumulated slowly from exome cohorts, but two recent, independent reports delineate monogenic hCTR1 deficiency as a distinct inherited disorder of copper metabolism. First, Batzios et al. [35] described families with biallelic likely pathogenic *SLC31A1* variants, defining a syndrome of early-onset neurodevelopmental impairment with biochemical evidence of systemic copper dyshomeostasis. These variants cause hypotonia, developmental delay, seizures, and low serum copper/ceruloplasmin, situating hCTR1 deficiency, similar to the effect of ATP7A mutations in Menkes disease on the copper-deficiency spectrum but upstream of ATP7A [35]. Mechanistically, these findings were recapitulated by mouse knockout experiments, which related to the discovery of hCTR1 [25,26]. Second, Dame et al. [217] reported a fatal congenital copper transport defect caused by a homozygous, likely pathogenic, *SLC31A1* variant, strengthening causality by linking genotype to a perinatal-lethal phenotype with copper-deficiency biochemistry. This leads to severe neonatal hypotonia, metabolic decompensation, and liver dysfunction, which is consistent with profound failure of copper entry as the primary disturbance. The defect arises before copper can load onto Atox1 and enter the secretory pathway or be exported, thereby phenocopying features of copper deficiency despite intact ATP7A/B coding sequence.

These human data frame *SLC31A1* as a third axis of inherited copper disease. Whereas Menkes/Wilson are copper routing or export disorders, hCTR1 deficiency is purely a copper import disorder. In practice, this has implications for biomarkers and therapy. For example, oral copper salts may be ineffective if apical uptake is severely compromised, suggesting that parenteral Cu formulations or strategies to bypass apical hCTR1 could be considered. Concepts familiar from Menkes management but here rationalized by a primary entry defect [193,197]. Finally, mechanistic ties between hCTR1 and CTR2 (biogenesis/stability), as well as copper-dependent endocytic regulation of hCTR1 (Section 4), may further modify phenotype when import is partially impaired [218].

### 5.3. hCTR1 and Cancer

Many tumors exhibit copper dependence for proliferation, redox buffering, and angiogenic signaling, making copper trafficking system part of the malignant metabolic program [13]. In various cancer types, hCTR1 is upregulated and is associated with prognosis in several solid tumors [219]. Additionally, recent knockdown studies of Atox1 in cancer cells demonstrated that this copper chaperone is crucial for cancer cell proliferation and survival [220,221,222]. Consistently, small molecules targeting Atox1 have been proved to effectively block Cu-trafficking and consequently reduce cell proliferation in lung, leukemia, breast, head, and neck cancer cell lines by elevating cellular ROS levels via Cu accumulation and reducing cellular NADPH and GSH levels. In contrast, healthy cells were barely affected by these small molecules [223]. Moreover, Atox1 and ATP7B were found to be important for Pt-based drug resistance. It was shown that by down-regulating levels of Atox1, Pt drug efflux was reduced, leading to increased Pt-induced cytotoxicity [224,225]. In a recent study, we showed that peptides that interact with Atox1 and MBD 3/4 units of ATP7B affect copper metabolism in breast and liver cancer cells and cause preferential toxicity in cancer cells [226]. Providing a promising avenue for novel therapeutics or targeted drug delivery systems.

**Figure 7:**
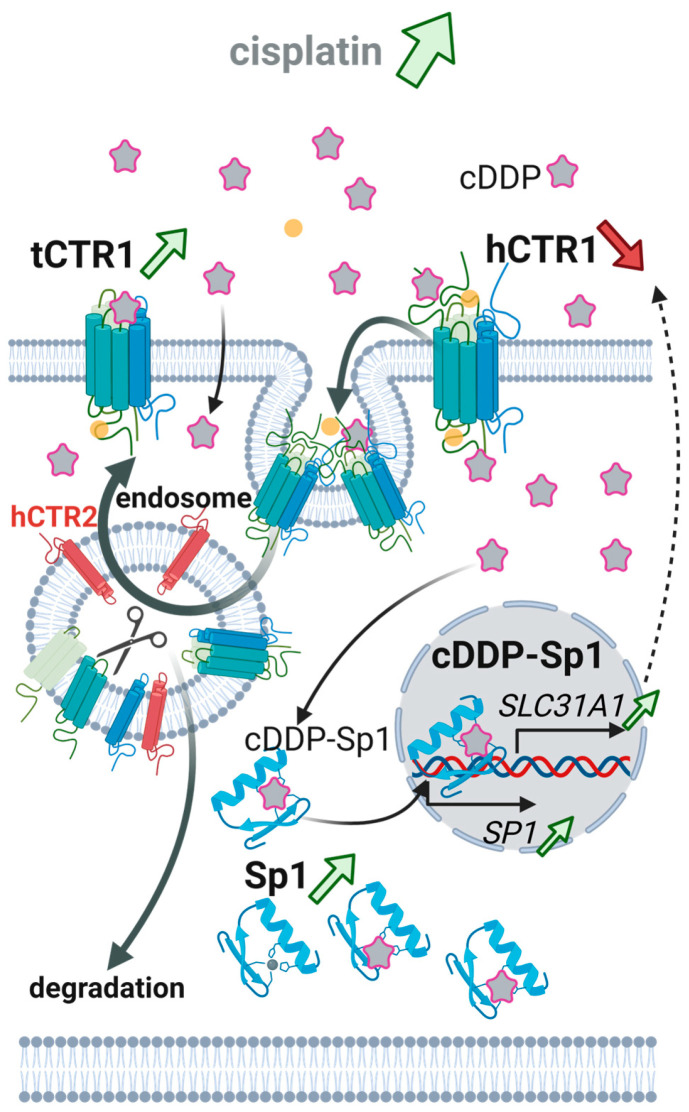
Uncoupling of hCTR1 Transcription and Protein Stability by cDDP. cDDP treatment mimics a copper-deficient state, triggering a compensatory transcriptional response via Sp1, which upregulates *SLC31A1* mRNA synthesis. Despite increased mRNA, functional surface hCTR1 protein levels decrease. Cisplatin triggers rapid internalization and ubiquitination of hCTR1, leading to proteolytic cleavage, truncated CTR1 (tCTR1) or proteasomal degradation. This post-translational degradation overrides the transcriptional increase, resulting in a net loss of drug uptake capacity.

The hCTR1-Atox1-ATP7B cycle is also linked to platinum chemotherapy. Cisplatin enters cells in part through hCTR1 showing that hCTR1 deletion may confer cDDP resistance and reduce intracellular platinum accumulation [39,227]. However, it must be noted that the entry route of cDDP is rather not exclusively through hCTR1 but rather a combination via a non-protein mediated route (Figure 7) [125]. Modern single-cell mass-spectrometry imaging confirms, at cellular resolution, that platinum accumulation tracks with hCTR1 localization, underscoring hCTR1 as a determinant of drug handling at the tumor cell surface [124]. Mechanistically, the abundance of full-length glycosylated hCTR1 protein decreases upon cDDP exposure due to enhanced proteolytic degradation (Figure 7) [109,148]. As stated in Section 4.1, hCTR1 is known to be proteolytically processed to a ~17 kDa truncated form in endo-lysosomal compartments upon elevated cDDP exposure [81]. Here cathepsin-dependent cleavage of the ectodomain limits both copper and cDDP acquisition [114]. Subsequently, CTR2 facilitates accumulation of the truncated form in the plasma membrane (Figure 7) [123]. In addition, it was shown that cDDP is facilitating Sp1 binding and therefore increases transcription of both *SP1* and *SLC31A* leading to increased levels of *SP1* and *SLC31A* mRNA [109].

In a clinical manner, hCTR1 is an attractive candidate biomarker for platinum sensitivity. In non-small cell lung cancer (NSCLC), higher tumoral hCTR1 expression has repeatedly correlated with increased intratumoral platinum accumulation, higher response rates and longer progression-free survival [147,228,229], and similar associations have been described in muscle-invasive bladder, ovarian and endometrial cancers treated with platinum-containing chemotherapy [230,231]. A recent pan-cancer TCGA analysis further identified SLC31A1 as a copper-related gene whose dysregulated expression and recurrent somatic mutations associate with prognosis and immune cell infiltration across multiple tumor types, supporting its broader potential as an oncologic biomarker and therapeutic target [232].

Nevertheless, hCTR1 has not yet entered routine clinical use and existing cohorts are relatively small, single-center and methodologically heterogeneous, no companion diagnostic has been approved, and to our knowledge current NCCN/ESMO guidelines do not recommend SLC31A1 testing for standard cDDP chemotherapy. Early-phase trials that combine platinum agents with copper-lowering drugs such as trientine have begun to translate hCTR1 biology into the clinic [233,234,235], but in these studies CTR1 has been evaluated only as a mechanistic or exploratory biomarker rather than a formal stratification factor. Accordingly, hCTR1/SLC31A1 currently remains an investigational predictive/prognostic marker, and prospective biomarker-driven trials with standardized hCTR1 assays will be needed before it can guide platinum therapy in routine practice.

Therapeutic opportunities emerge from this biology. Strategies include (i) preventing hCTR1 down-regulation during platinum therapy, (ii) copper chelation to upregulate/delocalize hCTR1 and sensitize tumors to Pt(II) drugs, or (iii) targeting hCTR1′s copper binding sites to pre-activate specific platinum prodrugs for enhanced import [38].

Finally, given the critical role of copper in cancer biology and cell death, hCTR1 expression, its associated chaperones, and intracellular trafficking should be assessed alongside ATP7A/B when interpreting tumor copper pharmacology or developing metal-based therapeutics. [192,236].

## 6. Outlook and Future Directions

HCTR1 plays a central role in maintaining copper homeostasis, a process essential for numerous cellular functions. Dysregulation of hCTR1 expression or activity has been linked to various diseases, particularly cancer and neurological disorders [35,39,237]. To fully exploit the biomedical potential of hCTR1, future research must address critical gaps in our understanding of the copper trafficking network. While Atox1 is well-characterized, the mechanisms and physiological roles of the copper chaperones CCS and COX17 remain incompletely understood [178,179,238]. Detailed elucidation of the molecular interplay between hCTR1 and these chaperones is crucial to clarify how copper is compartmentalized within cells, particularly regarding the regulation and interactions of the heterotrimeric hCTR1–CCS–SOD1 complex.

Moreover, the regulatory mechanisms governing hCTR1 require deeper investigation, especially the proteolytic cleavage of its N-terminal ectodomain. It is established that under conditions of elevated copper or cisplatin exposure, endo-lysosomal cathepsins cleave this domain [114]. The fate of the cleaved N-terminus remains an open question, though a leading hypothesis suggests that it is excised in a copper-bound (holo) state and subsequently excreted via lysosomes. This mechanism could provide cells with a rapid detoxification route, bypassing slower copper efflux pathways. Validating this model would not only deepen our understanding of copper homeostasis but also identify new therapeutic targets for modulating intracellular copper levels.

These molecular insights pave the way for innovative therapeutic strategies, particularly drug repurposing. Potential drugs which impact the copper homeostasis either chelate excess copper and facilitate its excretion or prevent its uptake through hCTR1 [51,239]. Such therapeutics therefore could target the N-terminal lumen of hCTR1 or directly block the transmembrane channel and prevent copper import [240]. A promising avenue would be to use small molecules that act as molecular glue between holo-hCTR1 C-terminus and its chaperones [241,242,243]. Such molecular glue would irreversibly bind the chaperones to hCTR1 and impair further copper trafficking through this channel. Another avenue is to target the transcription and translation levels of *SLC31A1* which results in lower copies of hCTR1 and reduced copper uptake [244]. A more elegant approach is to take advantage of hCTR1 endocytosis and use molecules which induce endocytosis thus reducing the copies of hCTR1 in the plasma membrane by its built-in regulation mechanism [245]. Also, here small molecules acting as molecular glue that induce clustering of hCTR1 could facilitate endocytosis of hCTR1 and lower the metal import into the cell. Existing copper-chelating agents (e.g., trientine, tetrathiomolybdate), originally approved for Wilson’s disease, are now being repurposed to lower systemic copper and upregulate hCTR1, thereby sensitizing resistant tumors to platinum-based chemotherapies [38,109,146,233,234,246,247,248]. Conversely, in neurodegenerative disorders such as Alzheimer’s disease, restoring copper balance may help mitigate oxidative stress [249]. Beyond synthetic drugs, there is growing interest in natural compounds and dietary phytochemicals [249]. Recent studies highlight the potential of polyphenols such as luteolin, quercetin, and apigenin [250,251,252]. Initially investigated for antitumoral effects and their ability to cross the blood–brain barrier, these compounds also exhibit intrinsic metal-chelating properties, offering a promising approach to restore copper homeostasis with favorable safety profiles. The therapeutic potential of these supplements for age-related diseases and cancer is supported by promising preclinical data, but further research and human clinical trials are necessary to confirm their efficacy and determine appropriate clinical use.

Advancing research on hCTR1 will require an integrated approach combining structural biology, molecular imaging, and pharmacogenomics to dissect its regulation and transport mechanisms [1,41]. These insights could drive the development of novel therapeutic and diagnostic compounds, ranging from repurposed chelators to targeted phytochemicals, that selectively modulate copper uptake, offering new strategies for treating, delaying, or even preventing cancer and neurological conditions.

## 7. Conclusions

Human CTR1 has evolved from its initial discovery as a genetic homolog into a cornerstone of cellular copper homeostasis. It functions as the primary high-affinity gateway for Cu(I) import and as a central hub for intracellular copper distribution. Its conserved homotrimeric architecture, methionine-rich selectivity filter, and multiple regulatory mechanisms, including transcriptional control, rapid endocytosis, and proteolytic cleavage, work together to ensure efficient uptake while preventing toxicity.

Nearly three decades of research have revealed important insights into its structure, dynamics, transport gating, and interactions with chaperones such as Atox1, COX17, and CCS. However, key questions remain unanswered. These include the molecular interfaces between hCTR1 and downstream pathways, the fate and function of cleaved N-terminal ectodomains, and the adaptive responses that occur under physiological and pathological stress. These unresolved mechanisms are central to understanding why copper homeostasis becomes dysregulated in a wide spectrum of disorders. hCTR1s involvement ranges from neurodegeneration and inherited copper syndromes, where it has been proposed as a “third axis” alongside Menkes and Wilson diseases, to cancer, where it affects the uptake and efficacy of platinum-based chemotherapies.

As the principal regulator of copper flux, hCTR1 represents an attractive therapeutic target. Modulating its expression, activity, or degradation offers routes to correct copper imbalances through repurposed chelators, small-molecule modulators, or phytochemicals. Continued integration of structural biology, molecular imaging, and system-level modeling will be essential to resolve the remaining mechanistic gaps. Ultimately, deeper insight into hCTR1 biology will support the development of novel diagnostic and therapeutic strategies across diverse human diseases.

## Data Availability

No new data were created or analyzed in this study. Data sharing is not applicable to this article.

## Abbreviations

The following abbreviations are used in this manuscript:

Aβ	Amyloid-β
AD	Alzheimer’s disease
ATP	Adenosine triphosphate
Atox1	Antioxidant protein 1
CCS	Copper Chaperone for Superoxide Dismutase
cDDP	cisplatin
COX	Cytochrome c oxidase copper chaperone
CTR1	copper transporter 1
EPAS1 (HIF2α)	Endothelial PAS domain-containing protein 1
EPR	Electron Paramagnetic Resonance
GSH	Glutathione
HD	Huntington’s disease
hCTR1	human CTR1
MBD	metal binding domain
MTF-1	Metal regulatory transcription factor 1
NADPH	Nicotinamide adenine dinucleotide phosphate
ROS	reactive oxygen species
SOD	Superoxide dismutase
Sp1	Transcription factor Sp1
tCTR1	truncated CTR1
TM	transmembrane
ZnT1	Zinc Transporter 1
